# Predictors of nonadherence to breast cancer screening guidelines in a United States urban comprehensive cancer center

**DOI:** 10.1002/cam4.6182

**Published:** 2023-06-17

**Authors:** Alexandra Wehbe, Madeleine R. Gonte, Suzanne C. O'Neill, Alit Amit‐Yousif, Kristen Purrington, Mark Manning, Michael S. Simon

**Affiliations:** ^1^ Harvard T.H. Chan School of Public Health Boston Massachusetts USA; ^2^ Department of Oncology Barbara Ann Karmanos Cancer Institute Detroit Michigan USA; ^3^ Population Studies and Disparities Research Program Barbara Ann Karmanos Cancer Institute Detroit Michigan USA; ^4^ Wayne State University School of Medicine Detroit Michigan USA; ^5^ Department of Oncology, Lombardi Cancer Center Georgetown University Washington DC USA; ^6^ Center for Breast Health Oakland Macomb Obstetrics and Gynecology Rochester Hills Michigan USA; ^7^ Department of Psychology Oakland University Rochester Michigan USA; ^8^ Center for Molecular Medicine and Genetics Wayne State University School of Medicine Detroit Michigan USA

**Keywords:** breast cancer, breast density, mammogram, screening

## Abstract

**Background:**

This study aimed to identify predictors of nonadherence to breast cancer screening guidelines in an urban screening clinic among high‐ and average‐risk women in the United States.

**Methods:**

We reviewed records of 6090 women who received ≥2 screening mammograms over 2 years at the Karmanos Cancer Institute to examine how breast cancer risk and breast density were associated with guideline‐concordant screening. Incongruent screening was defined as receiving supplemental imaging between screening mammograms for average‐risk women, and as *not* receiving recommended supplemental imaging for high‐risk women. We used *t*‐tests and chi‐square tests to examine bivariate associations with guideline‐congruent screening, and probit regression to regress guideline‐congruence unto breast cancer risk, breast density, and their interaction, controlling for age and race.

**Results:**

Incongruent screening was more likely among high‐ versus average‐risk women (97.7% vs. 0.9%, *p <* 0.01). Among average‐risk women, incongruent screening was more likely among those with dense versus nondense breasts (2.0% vs. 0.1%, *p* < 0.01). Among high‐risk women, incongruent screening was more likely among those with nondense versus dense breasts (99.5% vs. 95.2%, *p* < 0.01). The significant main effects of density and high‐risk on increased incongruent screening were qualified by a density by high‐risk interaction, showing a weaker association between risk and incongruent screening among women with dense breasts (simple slope = 3.71, *p* < 0.01) versus nondense breasts (simple slope = 5.79, *p* < 0.01). Age and race were not associated with incongruent screening.

**Conclusions:**

Lack of adherence to evidence‐based screening guidelines has led to underutilization of supplementary imaging for high‐risk women and potential overutilization for women with dense breasts without other risk factors.

## 1 INTRODUCTION

**FIGURE 1:**
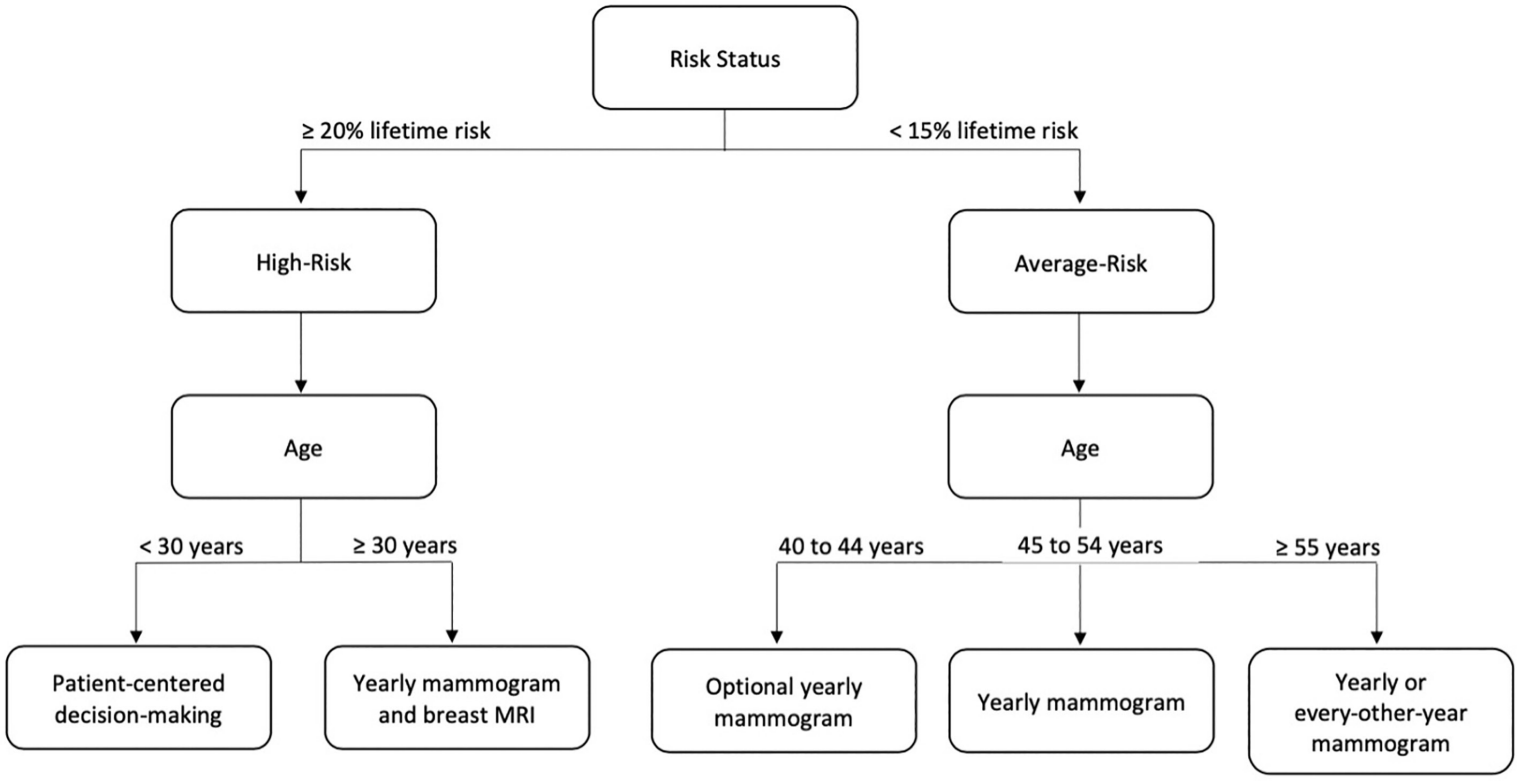
American Cancer Society recommendations for early detection of breast cancer based on risk status assessment.

Breast cancer screening has been shown to significantly reduce mortality due to breast cancer, and most breast cancers in the United States (US) are diagnosed due to an abnormal screening test.^1^, ^2^ The American Cancer Society (ACS) and several other US‐based professional health organizations have endorsed standardized screening as an integral component of secondary breast cancer prevention.^1^ Mammograms are the standard screening modality recommended by screening guidelines, with varying recommendations according to a patient's risk status and age (Figure 1).^3^ Findings suggest that adequate screening both reduces the odds of dying from breast cancer and enables early initiation of treatment.^4^ Additionally, supplemental screening with breast magnetic resonance imaging (MRI) has been shown to facilitate increased and early detection of breast cancer,^5^ and has been endorsed by a number of professional health societies (e.g., ACS,^2^ American College of Radiology,^6^ and National Comprehensive Cancer Network^7^) for breast cancer screening among high‐risk women since as early as 2007.^2^, ^8^ Specifically, the ACS recommends annual supplemental screening with breast MRI for women who are 30 years or older and at high‐risk for breast cancer (≥ 20% lifetime risk by the Tyrer‐Cuizik risk model)^8^; however, women who are at average‐risk for breast cancer (<15% lifetime risk) should not be offered supplemental screening, irrespective of age (Figure 1).^9^

The variable recommendations for breast cancer screening across numerous professional health organizations based on heterogenous risk assessment factors, has increased the complexity of breast screening for both women and providers. In the Breast Cancer Surveillance Consortium, a population‐based cohort study, Hill et al. found that only 6.3% of high‐risk women received supplemental screening with breast MRI in accordance with screening guidelines (guideline‐congruent).^10^ Barriers to screening identified in several studies exploring utilization of supplemental screening included lack of knowledge, low perceived risk of breast cancer, and low concern regarding breast cancer, as well as conflicting recommendations from primary care physicians.^10^ Further, breast cancer screening barriers disproportionately affect Black compared with White women.^11^ This is especially important given that Black women are at higher risk for breast cancer compared to White women and are more likely to be diagnosed at a more advanced stage.^12^ In addition, other studies have shown that Black women are less likely to perceive themselves at elevated risk for breast cancer,^13^ and in general have less knowledge about breast cancer risk factors and risk status, compared to White women.^14^

Supplemental screening of average‐risk women has been shown to increase false positive results,^15^ negatively impact psychological well‐being,^16^ and decrease the rate of follow‐up for subsequent guideline‐congruent breast cancer screening.^16^ In June 2015, a Breast Density Patient Notification law went into effect in the state of Michigan, requiring that physicians notify women who have heterogeneously or extremely dense breasts identified on mammography. It is important to note that this law did not make any recommendations regarding how breast density should inform breast cancer screening practices. Thereafter, studies have demonstrated up to a 10‐fold increase in supplemental breast cancer screening among average‐risk women with dense breasts, suggesting that women with dense breasts are more likely to be incongruently screened.^17^, ^18^, ^19^, ^20^, ^21^, ^22^, ^23^ Lower rates of guideline‐congruent screening have been reported among average‐risk Black women compared with their White counterparts.^22^

Whereas prior studies have evaluated potential predictors of supplemental breast cancer screening among high‐risk women recruited either from a high‐risk screening clinic,^24^, ^25^, ^26^ a research study sample,^5^ or a sample of women enlisted in the military,^27^ to our knowledge, only one other study has examined the use of supplemental breast cancer screening among high‐risk women from a general screening population.^10^ In this study, we report the rate of guideline‐congruent and ‐incongruent breast cancer screening behaviors among a sample of Black and White women who were part of a general screening population at a large comprehensive cancer center in Detroit, Michigan and report on predictors of congruent and incongruent screening. We hypothesize that there will be higher rates of incongruent breast cancer screening among high‐risk women, women with dense breasts, and Black women.

## 2 METHODS

### 2.1 Study population

Eligible participants included Black and White women who presented to the Karmanos Cancer Institute (KCI) for at least two screening mammograms between January 1, 2016 and March 31, 2018. 2D screening mammograms were offered to women with nondense breast tissue, and 3D screening mammograms were offered to all women with dense breasts at the KCI. We chose to include only Black and White patients in this analysis because they comprise the largest groups of women seen at the KCI mammography screening clinic. We chose the start date to obviate any effects of Michigan's recently adopted breast density notification law (June 2015) on supplemental breast cancer screening behaviors. The end date reflects the termination of use of mammography workflow software at the KCI which automatically estimated each patient's breast cancer risk score (PenRad Technologies). Women were excluded if they had any diagnostic breast imaging (with and without a subsequent biopsy) in the interval between their two mammograms or if they developed breast cancer during the two‐year study period. Women were also excluded if they had a prior history of invasive breast or ovarian cancer, ductal carcinoma in situ (DCIS), lobular carcinoma in situ (LCIS), or atypical ductal or lobular hyperplasia. Consequently, our dataset consisted of mammogram and supplemental breast imaging results specifically for screening purposes among asymptomatic women with no prior breast or ovarian cancer‐related history. Of note, this paper describes the proportion of women who received supplementary screening at the KCI and reflects referrals that could have been made by any of the participants' health care providers. Therefore, the referrals that occurred at the KCI mammography clinic do not represent a standard of care referral process developed at the KCI. The study was approved by the Wayne State University IRB.

### 2.2 Sample demographic and clinical factors

The age and race of women in the study population were extracted from clinic data. The age was divided by 10 and mean‐centered to facilitate analyses. The Tyrer‐Cuzick version 7 model of lifetime risk of breast cancer was included in the mammography report and was interpreted as an indicator of breast cancer risk for this analysis. The Tyrer‐Cuzick version 7 model is a risk assessment tool that incorporates the following variables to estimate lifetime risk of breast cancer: age, height, weight, age at menarche, parity, menopausal status, hormone therapy use, *BRCA* status, abnormal breast biopsy history, Ashkenazi inheritance, and family/personal history of breast cancer. Women were classified as high‐risk if their lifetime breast cancer risk was ≥20%; intermediate‐risk if their lifetime risk was between 15% and 20%; and average‐risk if their lifetime risk was <15%. Women who had a BI‐RADS Density classification of *C* or *D* (defined as heterogeneously dense or extremely dense, respectively) were classified as having dense breasts, whereas women with a BI‐RADS Density of *A* or *B* (almost entirely fatty tissue or scattered areas of fibroglandular density, respectively) were classified as having nondense breasts. Breast cancer risk and breast density data were recorded based on the most recent clinic encounter before the end of the study period.

### 2.3 Outcome measures

Women were classified as having supplemental breast screening if their clinic records indicated any breast imaging (i.e., MRI, ultrasound, and molecular breast imaging) for screening purposes in the interval between their screening mammograms. Whether women were undergoing diagnostic or screening mammograms was determined by whether the imaging appointment was categorized as diagnostic or screening within the electronic medical record system.

Since screening guidelines for intermediate‐risk women leave greater latitude for shared decision‐making regarding screening regimens, we restricted the current analysis to women with high‐ and average‐breast cancer risk so that we could more accurately determine whether breast cancer screening practices were congruent or incongruent with risk‐based screening guidelines. High‐risk women were classified as incongruent if they did not receive supplemental imaging between screening mammograms; average‐risk women were classified as incongruent if they received supplemental imaging between screening mammograms.

### 2.4 Statistical analysis

Using the full samples, we described the proportion of women who had supplemental breast cancer screening and examined the relationship between supplemental screening and potential explanatory factors including breast cancer risk, age at the time of the screening, and breast density using chi‐square and *t*‐tests. All analyses were performed using the SPSS version 25.

The analysis was restricted to high‐ and average‐risk women for whom we had data on breast density. We used a series of path analysis with probit regression to examine predictors of incongruent screening in three steps. In the first step, we regressed the indicator for incongruent screening for age at screening and racial group; and in the second step, we introduced the main effects of breast density and breast cancer risk (high vs. average as measured by the Tyrer‐Cuzick model). In the final step, we introduced a risk by density interaction to examine whether the effects of risk on incongruent screening were influenced by breast density. Models were fit with lavaan in R version 4.1.2.^28^ Given that 8% of our sample is over the age of 74 and screening MRI is not recommended per NCCN guidelines for high‐risk women over 75, we also conducted a sensitivity analysis excluding women over the age of 74 at the time of their screening exam.

## 3 RESULTS

Our sample consisted of 4462 Black and 1628 White women who had multiple screening events between January 1, 2016 and March 31, 2018. Most women (86.5%) were at average‐risk for breast cancer, while 7.7% were at intermediate‐risk, and 5.8% were at high‐risk. The clinical and demographic characteristics of the average‐ and high‐risk women in our sample are listed in Table 1. White women in the sample were more likely to be at high‐ (7.8% vs. 5.1%) and intermediate‐risk (10.7% vs. 6.6%) (χ^2^ (2) = 49.16, *p* < 0.01) and were also more likely to be older compared with Black women (62.53 vs. 60.51: *t*_6088_ = 7.07, *p* < 0.01). Overall, 23.8% of the women in the sample were identified by radiologists as having dense breasts. White women were more likely to have dense breasts compared to Black women (31.3% vs. 21.1%: χ^2^ (1) = 64.11, *p* < 0.01).

**TABLE 1:** Demographic and clinical characteristics comparing women who are at average‐ and high‐risk for breast cancer.

Characteristic	Total	Risk status	
		Average‐risk	High‐risk
Total (percent)		5269 (93.7%)	352 (6.3%)
Mean age at diagnosis (years)	61.1	62.1	52.2
Race (percent)			
Black	4462 (73.3%)	3943 (88.4%)	225 (5%)
White	1628 (26.7%)	1326 (81.4%)	127 (7.8%)
Breast density class			
A: Predominately fatty	1554 (26.9%)	1398 (90%)	64 (4.1%)
B: Scattered fibroglandular	2853 (49.3%)	2486 (87.1%)	143 (5%)
C: Heterogeneously dense	1265 (21.9%)	1040 (82.2%)	109 (8.6%)
D: Extremely dense	112 (1.9%)	83 (74.1%)	15 (13.4%)

Sixty‐one women (1%) in our sample had supplemental breast cancer screening. Of the women who received supplementary imaging, 32 were classified as having dense breasts; five were classified as having nondense breasts; and breast density data was missing for the 25 remaining women. Overall, women at high‐ (2.3%) and intermediate‐ (1.7%) risk were more likely to have supplemental breast cancer screening than women at average‐risk (0.9%; χ^2^(2) = 9.22, *p* = 0.01). Younger women (54.05 vs. 61.12; *t*_6088_ = 5.58, *p* < 0.01, *d* = 0.72) and women with dense breasts (2.3% vs. 0.1%; χ^2^ (1) = 80.65, *p* < 0.01) were also more likely to have supplemental breast cancer screening. There were no racial differences in the rate of supplemental screening.

### 3.1 Incongruent screening

Restricting the analysis to average‐ and high‐risk women, 390 (6.9%) of these women were incongruently screened which included 343 (97.7%) out of all high‐risk women and only 47 (0.9%) out of all average‐risk women (*p* = <0.01). Women were more likely to be incongruently screened if they were younger (52.69 vs. 62.18; *t*_5620_ = 18.79, *p* < 0.01, *d* = 0.98), had dense breasts (11.3% vs. 5.1%; χ^2^ (1) = 59.80, *p* < 0.01), or were White (9.2% vs. 6.1%; χ^2^(1) = 15.85, *p* < 0.01).

We also looked at factors associated with incongruent screening stratified by high‐ versus average‐risk status. Average‐risk women were more likely to be incongruently screened if they had dense breasts (2.0% with dense breast compared with 0.1% χ^2^ (1) = 62.09, *p* < 0.01) or if they were younger (average age 55.11 years vs. average age 62.20 years; t_5267_ = 4.87, *p* < 0.01). High‐risk women were more likely to be incongruently screened if they had nondense breasts (99.5% with nondense breasts vs. 95.2% with dense breasts; χ^2^ (1) = 7.04, *p* < 0.01), or if they were older (average age 52.38 years, vs. 47.50 years; t_351_ = 1.87, *p* = 0.06). There was no significant impact of race (Black vs. White) on the rate of incongruent screening for average‐ or high‐risk women.

### 3.2 Multivariate path analysis

Results of the analyses predicting incongruent screening for the entire cohort are presented in Table 2. Results at step 1 indicated that older women and Black women were less likely to be incongruently screened (*R* ^*2*^ = 1.64, *p* < 0.01); however, the effects of age and race were no longer significant with the addition or risk and high density in subsequent steps. The significant main effects of increased breast density and high‐risk status on a higher rate of incongruent screening as shown in step 2 were qualified by a density by high‐risk interaction indicated in the final model listed in step 3. Probing this interaction indicated that the association between breast cancer risk and incongruent screening was weaker among women with dense breasts (simple slope = 3.71, *R* ^*2*^ = 0.29, *p* < 0.01) compared with those without dense breasts (simple slope = 5.79, *R* ^*2*^ = 0.34, *p* < 0.01). In other words, women with dense breasts were less likely to be incongruently screened regardless of breast cancer risk. Examining the marginal probabilities consistent with the final model (Table 3), suggests that the weaker association between breast cancer risk and incongruent screening among women with dense breasts may be due to overscreening among average‐risk women. That is, the probability of incongruent screening for average‐risk women was higher for women with dense breasts compared with those without, thus attenuating the differences in the probabilities due to breast cancer risk for women with dense breasts compared with those without dense breasts. These results were unchanged in a sensitivity analysis excluding women over the age of 74.

**TABLE 2:** Stepwise analysis: predictors of incongruent supplemental screening.

	Step 1		Step 2		Step 3	
	b	SE	b	SE	b	SE
Predictors						
Age	−0.59**	0.04	−0.08	0.06	−0.09	0.07
Black	−0.39**	0.06	0.29	0.16	0.31	0.17
Dense	–	–	0.70**	0.19	0.08	0.24
High‐Risk	–	–	4.68**	0.18	4.75**	0.24
Dense*High‐risk	–	–	–	–	−2.07**	0.47
Threshold						
	1.64**	0.04	0.29*	0.12	0.34**	0.12
*R* ^*2*^						
Incongruent Screening	0.26		0.60		0.65	

*Note*: *p < 0.05; **p < 0.01; results at step 1 indicated that older women and Black women were less likely to be incongruently screened; however, the effect of age was rendered nonsignificant in subsequent steps. The significant main effects of breast density and high risk (step 2) were qualified by a density by high‐risk interaction indicated in the final model (step 3).

**TABLE 3:** Marginal probabilities of incongruent screening.

	Breast density	
	Dense	Not dense
Risk status		
Average‐risk	0.034	0.002
High‐risk	0.971	0.998

## 4 DISCUSSION

We examined predictors of guideline‐incongruent supplemental breast cancer screening among Black and White women at a large comprehensive cancer center screening clinic in Detroit, Michigan. Though only a small portion (1%) of women were supplementally screened, 6.9% of women who were either at average‐ or at high‐ risk for breast cancer had screening which was inconsistent with guidelines. Notably, 97.7% of high‐risk women in our sample were incongruently screened, meaning they did not receive supplementary screening when indicated. Guidelines indicate that high‐risk women should be screened with supplemental MRI in addition to screening mammogram,^2^, ^6^, ^7^, ^29^ and the benefits of early detection support supplemental screening for high‐risk women outweighs the potential harms of overdiagnoses and false positives.^30^, ^31^, ^32^ Nuances of breast cancer screening guidelines vary across organizations and are dynamic as new evidence is presented; nonetheless, the majority of high‐risk women in our sample (97.7%) did not receive supplemental screening, which is a violation of a well‐established guideline for high‐risk women.^1^, ^2^, ^7^, ^9^, ^33^, ^34^ Our results are consistent with prior studies, which have found underutilization of supplemental screening modalities, particularly MRI, among women who are at high risk for breast cancer, indicating potential underscreening for an at‐risk population of women.^35^ These findings are problematic, given that supplemental screening with MRI has been shown to increase cancer detection rates by up to 56% in high‐risk women.^30^, ^36^, ^37^, ^38^, ^39^, ^40^, ^41^, ^42^ However, while the ACS guidelines clearly state “Women who are at high risk for breast cancer based on certain factors should get a breast MRI and a mammogram every year, typically starting at age 30,” these guidelines also state “Most women at high risk should begin screening with MRI and mammograms when they are 30 and continue for as long as they are in good health. But this is a decision that should be made with a woman's health care providers, taking into account her personal circumstances and preferences.” This caveat may be a contributing factor to the nonreceipt of supplemental imaging by some women at high‐risk for breast cancer, as it may vary on a case‐by‐case basis. Further, the high rate of incongruent screening among women at high‐risk could be attributed to the lack of standardization of recommendations across guidelines from various organizations on management of women at high‐risk. While this study focuses on the ACS recommendations, other organizations such as the US Preventive Services Task Force do not comment on screening recommendations for women with a ≥ 20% lifetime risk of breast cancer, which may lead to varying recommendations based on which guidelines clinicians utilize when discerning appropriate screening practices.

Identifying barriers to guideline‐congruent screening among high‐risk women informs interventions to directly address inadequate supplementary screening management for at‐risk populations. The few studies that have explicitly examined barriers to supplemental screening among high‐risk women suggest that lack of knowledge (on the part of the patient, the physician, or both) and guideline‐inconsistent recommendations from their physicians contribute to low rates of supplemental screening.^25^, ^26^, ^27^ Furthermore, receipt of supplementary screening may also be hindered by high cost and lack of insurance coverage. For example, the preventative provision within the Affordable Care Act does not cover screening breast MRIs for women at high‐risk for breast cancer.^43^ Multiple modeling studies have found breast MRI to be 5–10 times more expensive than that of screening mammography.^43^, ^44^

Regarding knowledge, data from a sample of more than 11,000 women showed that knowledge of objective breast cancer risk factors are not associated with mammography screening attendance.^45^ However, subjective perceptions of breast cancer risk are positively associated with mammography screening.^46^ Given that high‐risk women are likely to underestimate their own breast cancer risk,^47^ some women at increased risk may not conceive of themselves as categorically high‐risk,^47^ resulting in breast cancer screening decisions that are inconsistent with their risk and incongruent with screening guidelines. Inconsistent guidance from physicians also contributes to poor compliance with screening recommendations. These barriers suggest the need for multilevel interventions that target both women's and physicians' knowledge, risk perceptions, and decision‐making.

Further, our multivariable analysis showed that women with dense breasts were less likely to be incongruently screened regardless of breast cancer risk. This weaker association between breast cancer risk and incongruent screening among women with dense breasts may be due to overscreening among average‐risk women with dense breasts. These findings support the ongoing uncertainty about recommendations for breast cancer screening for patients with dense breasts, despite increased density being an established risk factor. Given that there are currently no standardized guidelines directing management of breast cancer screening in the setting of dense breasts, clinicians may be inclined to recommend supplementary imaging regardless of recommendations since increased breast density is a known risk factor for breast cancer.

Guidelines also indicate that average‐risk women should refrain from supplemental screening unless clinically indicated^9^, ^33^, ^34^; however, results from our multivariable analysis show that average‐risk women with dense breasts had an increased likelihood of having supplementary screening compared with those without dense breasts. This could be attributed to mandated breast density notifications required by the 2015 breast density notification law in MI, which requires that notifications include the following statement: “…Dense breast tissue may increase your risk for breast cancer. This information about the result of your mammogram is given to you to raise your awareness. Use this information to discuss with your health care provider whether other supplemental tests in addition to your mammogram may be appropriate for you, based on your individual risk.”^17^, ^18^, ^19^, ^20^, ^21^, ^22^, ^23^ Thus, it is possible that some of the supplemental screening received by women at average‐risk for breast cancer could be partially attributed to shared decision‐making between the patient and clinician. Though this cannot be definitively discerned from our dataset, evidence suggests that physicians' supplemental screening referral practices diverged from recommended guidelines among recipients of density notifications.^48^ It is important to note, however, that the premise of the breast density notification law is to improve inadequate screening among women with dense breasts, as breast density is an independent risk factor for breast cancer. Some studies have asserted the benefits of supplemental screening in women with dense breasts. For example, the 2019 DENSE trial found that supplemental MRI screening in women with extremely dense breast tissue and negative screening mammography resulted in the diagnosis of significantly fewer interval cancers than mammography alone over 2 years.^49^

While increased breast density is an independent risk factor for breast cancer, the extent to which breast density affects risk for breast cancer is not absolutely established. Relative risk conferred by breast density in part depends on a woman's other risk factor patterns.^50^ Further, despite guidelines indicating that supplemental screening is not indicated, official recommendations are continuously evolving and there are not yet definitive trials that solidify whether supplementary screening is merited for women with dense breasts. Thus, applying expert or informed clinical opinion is not unreasonable in this scenario. While women with dense breasts were more likely to be incongruently screened regardless of risk, this weaker association between risk and incongruent screening among women with dense breasts could be partly due to the broader uncertainty of the role breast density plays in the breast cancer screening and risk that has not yet been captured in guideline recommendations.

Our study showed no racial differences in incongruent breast cancer screening in our study sample. This differs from prior studies which reported that Black women were more likely to be incongruently screened compared with their White counterparts following passage of density notification laws in MI.^22^ Other literature which suggests lower guideline‐congruent screening for Black women suggest that it is important to evaluate for racial disparities and it is also important to evaluate the role of referring physicians in screening decision, since women are likely to be screened in line with their physician's recommendation.^51^

### 4.1 Study limitations and strengths

These data are limited in that they originated from one screening center in an academic, metropolitan setting, which may limit generalizability. Nonetheless, as the screening clinic's population is majority Black, and represents the large and diverse screening population in the Metro Detroit, we feel that our results are at least generalizable to the population in the downtown KCI's catchment area. Our data did not account for variation due to physicians who referred women to the screening clinic for mammograms; therefore, the extent to which incongruent screening might be due to sociodemographic characteristics of referring physicians is unknown. Additionally, while supplementary screening for women at high‐risk for breast cancer has long been a well‐established guideline, it is important to note that any screening recommendation made by a clinician depends on a wide variety of factors not measured in this study, such as patient willingness to complete the imaging, social or financial barriers, or medical comorbidities which may influence whether a clinician thinks supplementary imaging is in the patient's best interest. Furthermore, our inclusion criteria may have resulted in some degree of selection bias in that we may have missed women who had a screening mammogram, followed by supplemental screening in the interval, but who did not return for a screening mammogram within the recommended two‐year interval (additional inclusion notwithstanding).

Future studies may account for physician characteristics and other sources of variation in women's screening behaviors (e.g., screening facilities, neighborhood‐level factors that affect access to care, and health insurers). Finally, we did not measure the women's perceptions of breast cancer risk, or attitudes toward supplemental screening; hence, potentially important individual‐level psychological predictors were absent in the data collected for this study. Despite these limitations, the demonstration of the scope of guideline‐incongruent screening among high‐risk women, and of the ostensible influence of breast density notifications on guideline‐incongruent screening among average‐risk women contribute to the strength of these data. Further, a major strength of our study is the large sample size of Black women compared to prior studies that assess the utilization of guideline‐concordant screening for breast cancer.^10^

## 5 CONCLUSION

Whereas health organizations promote evidence‐based guidelines for supplemental breast cancer screening in service of patient health, the lack of adherence to these guidelines is concerning. This concern is pressing for women who are at high‐risk for breast cancer since they were most likely to be incongruently screened, yet they are potentially most likely to benefit from the increased surveillance proffered by supplemental screening with MRI. These findings support the need for interventions to support guideline‐congruent behavioral decision‐making related to supplemental breast cancer screening so that we may ensure that women are more likely to partake in secondary screening measures in line with evidence‐based recommendations.

## AUTHOR CONTRIBUTIONS

**Alexandra Wehbe:** Conceptualization (lead); data curation (equal); formal analysis (equal); investigation (lead); methodology (equal); project administration (lead); resources (equal); software (equal); writing – original draft (lead); writing – review and editing (lead). **Madeleine Gonte:** Conceptualization (equal); investigation (equal); writing – original draft (equal); writing – review and editing (equal). **Suzanne C. O'Neill:** Conceptualization (equal); data curation (equal); investigation (equal); methodology (equal); writing – review and editing (equal). **Alit Amit‐Yousif:** Conceptualization (equal); data curation (equal); investigation (equal); methodology (equal); writing – review and editing (equal). **Kristen S. Purrington:** Conceptualization (equal); formal analysis (equal); methodology (equal); validation (equal); writing – review and editing (equal). **Mark Manning:** Conceptualization (equal); data curation (equal); formal analysis (lead); investigation (equal); methodology (equal); writing – original draft (equal); writing – review and editing (equal). **Michael Simon:** Conceptualization (equal); investigation (lead); supervision (lead); writing – review and editing (equal).

## FUNDING INFORMATION

There were no sources of funding.

## CONFLICT OF INTEREST STATEMENT

The authors have no conflicts of interest to disclose.

## ETHICS STATEMENT

This study was approved by the Institutional Review Board (IRB). According to IRB guidelines, neither approval from the ethics committee nor informed consent from the study populations is required for this study.

## PERMISSION TO REPRODUCE MATERIAL FROM OTHER SOURCES

This study did not use material from other sources.

## Data Availability

Data Availability Statement: The data that support the findings of this study are available from the corresponding author upon reasonable request.
